# Through Thick and Thin: Changes in Creativity During the First Lockdown of the COVID-19 Pandemic

**DOI:** 10.3389/fpsyg.2022.821550

**Published:** 2022-05-10

**Authors:** Alizée Lopez-Persem, Théophile Bieth, Stella Guiet, Marcela Ovando-Tellez, Emmanuelle Volle

**Affiliations:** ^1^FrontLab, Sorbonne Université, Institut du Cerveau - Paris Brain Institute - ICM, INSERM, CNRS, AP-HP, Hôpital de la Pitié Salpêtrière, Paris, France; ^2^Neurology Department, AP-HP, Hôpital de la Pitié Salpêtrière, Paris, France

**Keywords:** creativity, COVID-19, survey, lockdown, creative activities

## Abstract

COVID-19 took us by surprise. We all had to face the lockdown and pandemic that put us in a new context, changing our way of life, work conditions, and habits. Coping with such an unprecedented situation may have stimulated creativity. However, the situation also restricted our liberties and triggered health or psychological difficulties. We carried out an online survey (*n* = 380) to examine whether and how the COVID-19 related first lockdown period was associated with creativity changes in French speaking population. Despite a global negative subjective experience of the situation, participants reported that they were more creative during the lockdown than before. Positive changes were linked with more time availability, more motivation, or the need to solve a problem while negative changes were related to negative affective feelings or a lack of resources or opportunities. This study documents the effects of the first lockdown period on creativity and the factors that influenced it.

## Introduction

The context of the COVID-19 pandemic has led to the implementation of unprecedented restrictive measures in society. During spring 2020, 67 million French people entered the first strict lockdown, which dramatically changed our daily lives and wellbeing. These constraints implied a restriction of movement, the need to adapt working methods, a modification of daily life within households, and a decreased wellbeing (linked to health issues, restrictions of activities and social interactions, or economic consequences). Paradoxically, during this period, general and social media have reported that individuals and businesses seemed to show great adaptability to face the situation by finding innovative solutions and creative behaviors.

Creativity is defined in science as the ability to produce something that is both original and appropriate (Lubart et al., 2013). Creative abilities are involved in many human activities, from solving everyday problems to the great discoveries of our civilization. Creativity is a key mental capacity to cope with change, invent and innovate, and face the challenges of our society. The situation related to the COVID-19 and its consequences on our daily life are at the forefront of the current challenges we had and still have to cope with. Understanding the conditions that foster (or hinder) our creativity is essential, and the COVID-19 situation provides a unique opportunity to study some of these factors.

According to the multidimensional approach to creativity (Lubart et al., 2015), creativity and creative behaviors rely on cognitive factors (our mental capacities) and individual personality. It is influenced by internal and external factors (Lubart et al., 2013) including our emotions, motivation, and environment. Intrinsic motivation is an essential component of creative behavior (Amabile, 1983, 2018; Amabile and Pillemer, 2012; Mastria et al., 2018; Benedek et al., 2019; Fischer et al., 2019) and drives involvement, intensity, and perseverance in activities. Affective states influence creativity, as it has been demonstrated by several studies which have shown that creative thinking tasks are mood-sensitive (Davis, 2009). Positive mood (such as joy) is often reported as facilitating creativity, and negative mood (such as stress, anxiety) as hindering creativity. However, whether positive or negative moods facilitate or inhibit creativity is still debated and may depend on contextual factors (Baas et al., 2008; De Dreu et al., 2008; Davis, 2009). Finally, creative activities and achievements are influenced by our environment, at home or in the workspace (living conditions, cultural, and professional contexts), our social context (Amabile and Pillemer, 2012; Benedek et al., 2019), and by the standards, needs, and values of the society (Lubart et al., 2015).

The situation related to COVID-19 likely had a major impact on many if not all the creativity dimensions or factors described above, by affecting our emotions, motivations, and wellbeing (Bu et al., 2020; Li et al., 2020; Michinov and Michinov, 2020; Roma et al., 2020; Zabelina et al., 2021), modifying our environment, our lifestyle, and availability, but also the standards and incentives of our society during this period, opening a new framework of thought (Kapoor and Kaufman, 2020; Beghetto, 2021). The few existing scientific studies that explored creativity during the COVID-19 pandemic have indeed suggested that the lockdown period facilitated everyday creativity (Karwowski et al., 2021; Mercier et al., 2021) or that creative abilities could help people dealing with the situation and improve their wellbeing (Michinov and Michinov, 2020; Tang et al., 2020; Elisondo, 2021; Hofreiter et al., 2021; Orkibi, 2021). However, those studies did not assess qualitative and quantitative aspects of how creativity changes related to environmental, contextual, or emotional changes.

The current study aimed at exploring how the lockdown and context related to COVID-19 impacted creativity and identifying the potential factors linked to these changes. We assumed that the lockdown could have stimulated creativity for at least two reasons. First, the lockdown increased the availability of free time by reducing our usual daily outdoor activities and, in some cases, workload. Second, the new situation created a need or drive, pushing the individuals to adapt and invent new solutions to pursue their usual activities. However, the situation may have caused negative subjective experience and distress, with stress and anxiety, a feeling of pressure, a lower mood, which can alter creativity. Last, because the restrictive measures targeted social interactions, we hypothesized that social distancing could also have harmed creativity. We carried out an online survey to estimate the impact of these potential factors on self-perceived creativity changes and changes in concrete creative activities and achievements.

## Materials and Methods

### Ethics and Participants

The study was approved by an ethical committee. All the data were collected anonymously online and in compliance with the General Data Protection Regulation (GDPR). The participants were recruited online through social networks. Respondents were invited to complete the entire survey if they were French speakers over 18 years old, agreed to participate, and did not already participate.

In total, we collected data from 551 participants (365 females and 186 males; mean age 42.31 years, age ranging from 18 to 84 years). Participants were French speakers and completed the survey between May 27th and August 1st of the year 2020. All questions referred to the lockdown period, that was, in mainland France, from March 16th to May 11th of the year 2020. Data from 171 participants were removed because they did not complete more than 50% of the survey. The final sample of the survey considered the data collected from 380 participants (281 females and 99 males; mean age 43.16 years, age ranging from 18 to 84 years). All demographic details about included participants are provided in Supplementary Method 1, Table 1, and Supplementary Table 1.

**TABLE 1:** Demographic data.

Participants	Total included		SCC Mean	SCC SEM
	380		8.0	0.98

**Gender [*F*(1, 378) = 0.01, *p* = 0.93]**	***N***	**%**	**SCC Mean**	**SCC SEM**

Female	281	73.95	8.12	1.17
Male	99	26.05	7.94	1.78

**Education [rho(378) = 0.04, *p* = 0.44]**	***N***	**%**	**SCC Mean**	**SCC SEM**

No High School Diploma	13	3.42	0.15	5.91
High School Diploma or Secondary Education Certificate (Baccalaureat in French)	32	8.42	9.56	3.64
2 or 3 years completed after High School Diploma (e.g., Bachelor’s degree)	73	19.21	7.78	2.17
4 or 5 years completed after High School Diploma (e.g., Master’s degree)	154	40.53	7.6	1.61
More than 6 years completed after High School Diploma (e.g., Doctorate’s degree)	108	28.42	9.46	1.66

**Age (mean = 43.16, *SE* = 15.85) [*r*(378) = −0.02, *p* = 0.69, (continuous measure)]**	***N***	**%**	**SCC Mean**	**SCC SEM**

18–21	8	2.11	5.75	6.08
21–30	97	25.53	9.98	1.89
31–40	79	20.79	6.94	2.22
41–50	66	17.37	4.73	2.25
51–60	76	20.00	11.42	1.98
61–70	30	7.89	7.63	4.31
71 +	20	5.26	3.7	4.62

**Residential area [*F*(2, 377) = 0.43, *p* = 0.65]**	***N***	**%**	**SCC Mean**	**SCC SEM**

Urban	191	50.26	8.4	1.27
Peri	128	33.68	6.92	1.85
Rural	61	16.05	9.49	2.51

**Residence during lockdown [*F*(1, 378) = 0, *p* = 0.99]**	***N***	**%**	**SCC Mean**	**SCC SEM**

My home	322	84.74	8.07	01.05
Other	58	15.26	8.1	2.64

**Country [*F*(5, 317) = 0.25, *p* = 0.94]**	***N***	**%**	**SCC Mean**	**SCC SEM**

France	293	77.11	7.9	1.12
France-outre mer	2	0.53	8.5	8.5
Suisse	2	0.53	18	2
Canada	2	0.53	2	8
Belgique	6	1.58	8.67	4.2
Other	18	4.74	9.67	4.9

**Professional activity [*F*(1, 378) = 0, *p* = 0.99]**	***N***	**%**	**SCC Mean**	**SCC SEM**

Related to the fight against COVID-19	43	11.32	7.86	3.15
Unrelated to the fight against COVID-19	315	82.89	7.87	1.06

*Each information is displayed with statistical tests (one-factor ANOVA for categorial factors, Spearman correlation coefficients for ordinal factors, Pearson correlation coefficients for continuous factors) on SCC. SCC mean and SEM are reported in the table. N indicates the number of participants in each category.*

### Survey and Scores

The survey was programmed in Qualtrics as a self-administered questionnaire. We collected four main categories of information including demographic data, several creativity measures, physical conditions and affective experience of the lockdown with independent questions (see Supplementary Method 1). The collected information is detailed below and the content of the survey is further detailed in Supplementary Tables 1–4.

#### Demographic Data and Lockdown Situation and Residency

We collected individual basic demographic information including age, gender, education level, socio-professional activity, and main field of activity. We also collected more specific information relative to the lockdown residency (residential area type, own residence, country of residence, access to private or large public outdoor spaces), whether professional activity was COVID-19 related, professional situation, and the occurrence of a serious problem that could have limited the initiation of activities (and in this case, how long it limited one’s activities) (Supplementary Table 1). To ensure that our results were not impacted by participants living outside of France (with potential different lockdown dates and restrictions), we re-ran our main analyses with participants who indicated living in France (*n* = 293) and found that all results remained qualitatively unchanged.

#### Measures of Creativity

We assessed creativity using four different approaches related to participants’ creativity.

##### Subjective Creativity Change

The main measure was the ***subjective creativity change (SCC)*** that aims to capture the self-perceived creativity change during the lockdown period. We asked participants how much they think their creativity changed positively or negatively during the lockdown as compared to before. The participants responded using a continuous visual scale ranging from −50 (less creative during the lockdown) to 50 (more creative during the lockdown). Hence, *SCC* relies on a self-rating of one’s own creativity. Similar self-report scales or ratings measuring creativity belief, self-concept, and self-efficacy have been developed (Beghetto, 2006; Ng and Feldman, 2012; Karwowski and Barbot, 2016; Karwowski and Lebuda, 2016, 2017; Karwowski et al., 2019) and used in COVID-19-related studies (Hofreiter et al., 2021; Mercier et al., 2021; Patston et al., 2021). In contrast to these previous measures, we asked participants to rate a change in their creativity related to a key event (i.e., the beginning of the lockdown). This allowed us to minimize recall bias and avoid the need of baseline measure that was difficult to obtain. To check our approach’s validity, we compared this new creativity measure to more standard ones, including openness personality traits and several activity-based measures described below.

##### Openness Personality Traits

We adapted a French-translated version of the ***Openness part of the Big Five Inventory (BFI-O)*** (Plaisant et al., 2010). This is an 11 questions questionnaire that evaluates the openness personality traits of participants. We chose the *BFI-O* because openness is the personality trait most associated with creativity (Beaty et al., 2017; Vartanian et al., 2018). The participants answered each of the 11 questions using a 5-point Likert scale, converted to individual scores ranging from −2 to 2. The *BFI-O* score was computed as the mean of the 11 individual responses recorded in the *BFI-O*.

##### Changes in Creative Activities and Achievements, and Reasons for Changes

In addition, we collected information about lockdown-related changes concerning participants’ involvement in creative activities in an independent part of the survey. We aimed to qualitatively and quantitatively assess the creativity changes induced by the lockdown on a list of 28 creative activities (see Supplementary Table 2). The 28 activities were selected based on existing items in other validated creativity questionnaires, including Inventory of Creative Activities and Achievements (ICAA) (Diedrich et al., 2017), Creative Behavior Inventory (CBI) (Hocevar, 1979), and Runco Ideational Behavioral Scale (RIBS) (Runco et al., 2014). For each activity, participants had to answer sequential questions about their involvement in this activity. The sequence of questions was conditional to their previous responses as the survey progressed (see Supplementary Figure 1 for a summary). The sequence was repeated for each of the 28 activities as follows.

For each activity, participants were first asked whether they had performed the activity during the last 5 years (lockdown period included). If not, the sequence stopped, and they were asked about the next activity. If so, they were asked whether they did it during the lockdown period and at which frequency (“much less,” “less,” “as much,” “more,” or “much more” than before the lockdown period). They could also indicate whether they did it only during the lockdown period, i.e., if the activity was new for them. The responses were converted into a ranking value from −2 (“much less”) to 2 (“much more”) providing an ***activity frequency change score***. In case the activity was not performed during the lockdown period, we assigned the value of −3. The frequency change score was computed for each participant as the mean of these ranking values across activities. We also computed the proportion of activities for each participant for which there was a negative, a positive, or no change.

In the next step, we aimed to understand the reasons for the changes. For each activity that was reported either less or more performed during the lockdown period than before, multiple-choice questions were displayed with several potential reasons for the change and the participants selected the ones that applied to them (different options were proposed for negative and positive changes, see Supplementary Table 3). Participants also had the option to write down their answers if none of the options were satisfying. We manually reviewed the responses they provided, and based on the frequency of some of them, we created two new *a posteriori* categories of motives for positive changes (see Supplementary Table 3). For each participant, and separately for positive and negative changes, we computed the number of times each reason was selected divided by the total number of activities for which changes were reported. This allowed us to quantify the main reasons leading to positive and negative changes in creative activities.

Finally, for activities performed as much or more often during the lockdown period than before, we asked: (1) How participants valorized it: Participants were asked to make a multiple choice among 11 options to assess the level of achievement of the performed activity (see Supplementary Table 3). Using a procedure similar to the ICAA scoring, each option was converted to a numerical value and the values corresponding to all the selected options for a given activity were summed. If participants shared their creative activities at least beyond the immediate surroundings (friends, family, cohabitant, or colleague), they automatically reached 8 points, and the three first proposals were not considered in the sum. Then, participant’s scores were averaged across activities for each participant to compute an individual ***creative achievement score***. (2) To report their feeling of having encountered obstacles during its realization using a visual quasi-continuous scale of 101 values from 0 (no obstacles) to 100 (many obstacles). The ***obstacle score*** was computed as the mean of obstacles rating across activities for each participant.

When the series of questions related to one activity (and dependent on participant’s responses) was complete, the same sequence was proposed for the next activity until the end of the list of 28 activities.

##### Creativity Rating

Finally, at the end of the survey, we asked participants to freely report the five most creative realizations (or top-productions) they carried out during the lockdown. They entered their responses directly in a text box without any time or word limit and with the possibility to leave empty boxes. An external panel of four experts independently rated the creativity of each of the top-productions using a 5-point Likert scale from 0 (“not creative”) to 5 (“highly creative”). The number of four raters have been previously suggested as sufficient to reach high inter-rater reliability when raters are experts (interrater reliability higher than 0.6) (Ceh et al., 2021). We estimated the interrater reliability by computing two-way random intra class correlations (ICC). A satisfying reliability was observed between judges. The average measure ICC was 0.95 [*F*(286, 858) = 19.84, *p* < 0.001, 95% CI (0.94.A200.96)]. Then, the ratings were averaged across judges for each top-production, and we computed for each participant a ***creativity rating score*** as the sum of averaged ratings of his/her productions. The *creativity rating score* aims to reflect the creativity of the activities achieved during the lockdown. Hence, it may both depend on the pre-existing creativity of the participants and on the effect of the lockdown situation on their creative thinking and behavior.

#### Psychological and Physical Conditions of Lockdown

In the survey, we asked specific questions about how participants experienced the lockdown. The aim was to identify subjective and objective factors that can explain creativity changes based on our hypotheses. We collected four categories of information.

##### Environmental Conditions

We asked the participants to report the number of cohabitants and the number of available rooms in the residence place, allowing us to compute the objective available ***space per cohabitant*** (i.e., the number of rooms per inhabitant). We also collected the subjective feeling of change in ***physical constraints***.

##### Social Relationships

Participants were asked whether they exchanged with as many people during the lockdown period as before to estimate changes in ***social interactions*** and the feeling of a change in ***loneliness***. These variables aim to reflect changes in social relationships during the lockdown period.

##### Available Time

Participants were asked to report the objective ***number of working hours*** per week (multiple-choice question with eight options ranging from “not concerned” to “more than 42 h” with an increment of 7 h per option) and the feeling of a change in ***free time***.

##### Affective Changes

Affective changes were assessed by asking participants their feeling of a change in ***mood, motivation, anxiety and stress, and pressure*** using separate questions.

All the questions focused on a change refer to a difference between the period before and during the lockdown. All subjective information was collected using multiple-choice questions with eight available choices: seven ranging from “much less” to “much more” and one allowing a neutral response (“I do not know”). Change in *free time* and in *social interactions* had only five available choices ranging from “much less” to “much more.” Those variables were considered as continuous (visual scales) or ordinal (Likert scales), and the sign was reversed for the ones with a negative meaning (indicated with “R” in figures and tables, such as *loneliness*) in order to obtain homogeneous valence across them (the higher, the better).

### Statistical Analyses

We provide a demographical description of our sample using percentage relative to the total number of participants for each demographic variable. To explore how the participants experienced the lockdown, we tested the difference from zero for significance at the group level using one-sample two-tailed *t*-tests, where independent variables were the subjective feeling of change in environmental conditions, social relationships, available time, and affects (see above).

We explored whether the lockdown impacted the subjective creative change (measured as *SCC*) using a one-sample two-tailed *t*-test against zero at the group level. To estimate the impact of the four hypothesized factors (environmental conditions, social relationships, time availability, affective experience) on SCC, we first conducted a principal component analysis (PCA). It reduced the ten variables used (i.e., *space per cohabitant*, *physical constraints*, *social interactions*, *loneliness*, *number of working hours*, *free time*, *mood*, *motivation*, *anxiety and stress*, and *pressure)* into a smaller set of dimensions while retaining as much of the original information as possible. It also helped to deal with the problem of multicollinearity among our variables. We ran the PCA on our ten variables of interest with oblique rotation (direct oblimin) to rotate loadings and identify the variables contributing the most to each component, using the available complete data from 293 participants. Factors with eigenvalues over Kaiser’s criterion of 1 were kept. Sampling adequacy for the analysis was assessed with the Kaiser–Meyer–Olkin measure (standard threshold = 0.5). To identify potential links between factors and change in creativity, correlation analyses were performed using Pearson coefficient between *SCC* score and the component loadings across subjects.

Additionally, we checked whether demographical information influenced *SCC*. For binomial and categorical variables, one-way analyses of variance (ANOVAs) were used to test their effect on *SCC*. Correlation analyses were performed using Pearson coefficient (r) for continuous variables (such as age) and Spearman coefficient (rho) for ordinal variables (such as education).

We also explored whether the relationship between *creativity rating score* and *SCC* could be hypothetically mediated by creative personality traits (*BFI-O*) or by factors that were found to influence creativity. We ran mediation analyses. We tested whether the direct path from *creativity rating score* to *SCC* was no longer significant when introducing the mediator M (β*_SCC–*RS*,_ M being either the *BFI-O* or the significant factor). Thus, the mediation analysis was estimated using the two following linear models:


SCC=β×SCC-creativityratingscorecreativityratingscore

SCC=β*×SCC-creativityratingscorecreativityratingscore+β×SCC-MM

Path significance (β_*SCC*–_*_*creativity rating score*_* −β*_SCC–_*_*creativity rating score*_*) was then assessed with a bootstrap test. We reported the 95 percent bootstrap confidence interval of the difference in regression coefficients between the direct and indirect path computed for 5,000 bootsamples. The mediation remains significant if this confidence interval does not include 0.

The same method was applied to investigate the relationship between *BFI-O*, *SCC* and Component 1.

With activity-based measures, our measures of interests were: (1) the five activities with the larger increase and decrease in frequency during the lockdown; (2) the categories of reasons explaining positive or negative changes in creative engagement; (3) the feeling of having encountered obstacles during its realization (*obstacle score*); (4) the way participants valorized their creative productions (*creative achievement score*).

We checked for consistency between all variables of interest. First, we explored the consistency of subjective creativity measure (*SCC*) with other measures of creativity using Pearson coefficient correlations (r) for continuous variables (*BFI-O* and *creativity rating score*) or with Spearman coefficient correlation (rho) for ordinal variables (*activity frequency change score, creative achievement score, obstacle score).* As we hypothesized that the amount of encountered obstacles could positively or negatively impact creativity, we also conducted a second order polynomial regression between SCC and *obstacle score*, as stated below:


O=β+0βSlinCC+βSquadrCC2

A significant quadratic term indicates that obstacles affect creativity both positively and negatively (fewer obstacles would have a lower effect).

Second, we explored the consistency between reasons explaining changes in engaging in specific activities and the general subjective experience of the lockdown. We ran correlation analyses between the proportions of the main activity-based selected reasons (that were related to free time and affective states) and the components of our previous PCA that related to changes in *SCC*. For activity-related reasons, we computed the difference in the percentage of positive and negative reasons as follows: Free time-related reasons were the difference between the proportion of “More Time” and of “No time.” Affective change-related reasons were the difference in proportion between “Inspired” and both “Concerned and worries”/“Did not feel like it.” The difference between positive and negative reasons was computed as they might capture the same effect. For example, if an individual selects “More time” as often as “No time” across activities (scores around zero), it might reflect a small change in experienced free time.

Third, we explore the consistency between within-factor measures of general subjective experiences of the lockdown. When relevant, we used Spearman (r) or Pearson (rho) coefficient correlation between factors that captured similar information (such as between *physical constraints* and *space per cohabitant*).

Finally, to check the validity of the list of activities used in the activity-based measures, we examined whether each of the top-productions corresponded to an activity included in our list. We provided the mean creative rating score for those not included in our list and specified what type of activity it was. We also checked if the most frequent productions were in line with the five activities with the highest increase in frequency in the activity-based measures.

All analyses were performed using Matlab Statistical Toolbox (Matlab R2020a, The MathWorks, Inc., United States), SPSS (v22.0; IBM Corp.), and R (v3.6.2).

## Results

### Demographics and Lockdown Situation

Overall, the majority of participants (*n* = 380) were confined in France (77%, and about 13% did not respond to that question), at home (85%), in urban areas (50%). 63% had access to private or large public outdoor spaces, 60% were confined with one or more individuals, and 87% declared not having encountered a severe problem during the lockdown. 54% were working remotely, and 83% had a professional activity not related to the fight against the pandemic. All demographic details are provided in Table 1 and Supplementary Table 1.

### Living Conditions and Subjective Experience of the Lockdown Situation

In the survey, participants were asked subjective and objective questions to gather information about their lockdown experience. As expected, on average and in terms of subjective experience, our sample felt more physically constrained [*physical constraints*: *M* = 0.33 ± 0.08 (SEM, Standard Error of the Mean), *t*(379) = 4.16, *p* < 0.001], more lonely [*loneliness*: *M* = 0.32 ± 0.07 (SEM), *t*(379) = 4.69, *p* < 0.001], and with more free time [*free time*: *M* = 0.6 ± 0.07 (SEM), *t*(379) = 8.82, *p* < 0.001]. In terms of affective changes, they felt more anxious or stressed [*anxiety and stress*: *M* = 0.32 ± 0.08 (SEM), *t*(379) = 3.96, *p* < 0.001], and had a poorer mood [*mood*: *M* = −0.18 ± 0.07 (SEM), *t*(379) = −2.39, *p* = 0.02]. On average, they did not feel significantly more or less motivated [*motivation*: *M* = 0.12 ± 0.09 (SEM), *t*(379) = 1.39, *p* = 0.17], nor experienced more (or less) psychological pressure [*pressure*: *M* = 0.09 ± 0.09 (SEM), *t*(379) = 1, *p* = 0.32].

Within-factor measures were consistent. Indeed, the subjective report of change in *physical constraints* was correlated with the *space per cohabitant* [M(space per cohabitant) = 1.19 ± 0.03 (SEM); *r*(378) = 0.10, *p* = 0.049], the subjective report of change in *loneliness* was correlated with the quantitative change in *social interactions* [M(change in interactions) = −0.22 ± 0.07 (SEM), *t*(379) = −3.31, *p* = 0.001; *r*(378) = 0.18, *p* = 0.005]. The subjective report of change in *free time* was correlated with the *number of working hours* per week (20% worked more than 42 h/week, 19% between 36 and 42 h/week, 15% between 29 and 35 h/week, 13% between 22 and 28 h/week, 14% between 15 and 21 h/week, 9% between 8 and 14 h/week, 10% less than 7 h/week, *r*(291) = 0.53, *p* < 0.001). Additionally, affective changes in *mood*, *motivation*, *anxiety and stress*, and *pressure* were all correlated to each other (all *p* < 0.001).

### Subjective Creativity Changes During the Lockdown

#### Measures of Creativity

**FIGURE 1:**
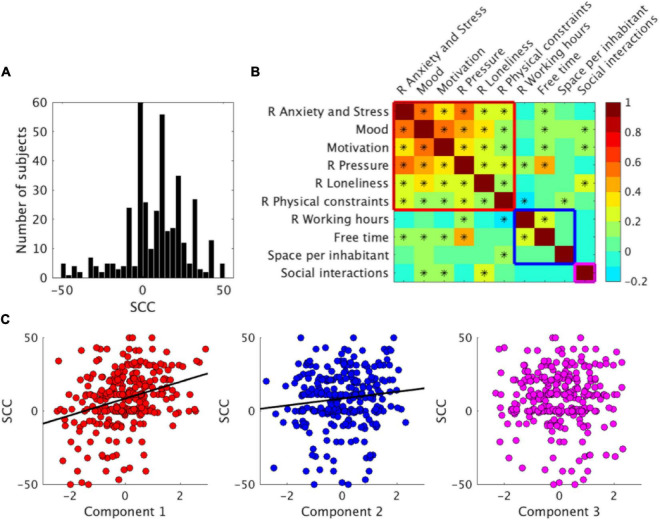
*Subjective creativity change (SCC)* and subjective experience of the lockdown. **(A)** Histogram of *SCC* scores. **(B)** Correlation matrix between measures assessing the living conditions and subjective experience of the lockdown situation. Stars indicate *p* < 0.05. Cold to hot colors represent the Pearson correlation coefficient (r). R indicates that the measure’s sign was reversed for positive values as positive changes (improvement) for all variables. Colored squares group measures clustering on the three principal components (red: Component 1, blue: Component 2, magenta: Component 3). **(C)** *SCC* as a function of participant’s component loadings. One dot represents one participant, and bold lines indicate that the correlation between the principal component and *SCC* was significant (Pearson correlation, *p* < 0.05).

When prompted to subjectively report how much they think their creativity changed during the lockdown (*SCC*, rating scale from −50 to 50), the participants declared on average to have been more creative during the lockdown period [*M* = 8.08 ± 0.98 (SEM), *t*(379) = 8.26, *p* < 0.001] (Figure 1A).

Additionally, we collected two other measures of creativity, that were the *creativity rating score* (corresponding to the external creativity rating of the five most creative productions the participants carried out during the lockdown) and the *BFI-O* (assessing creative personality traits). *Creativity rating score* mean was 7.74 ± 0.21 (SEM) across the 286 participants who responded to that question, and *BFI-O* questionnaire was on average 3.68 ± 0.03 (SEM). The *BFI-O* scores, *creativity rating score* and *SCC* were all significantly pairwise correlated [*r_*BFI*–*O*_*_–_*_*creativity rating score*_*(378) = 0.46, *p* < 0.001, *r_*BFI*–*O*_*_–_*_*SCC*_*(378) = 0.21, *p* = 0.03, *r_*SCC*_*_–_*_*creativity rating score*_*(378) = 0.15, *p* = 0.009]. Hence, as the *creativity rating score* might reflect both the creative personality traits and creativity changes during the lockdown, we tested whether *BFI-O* scores could be a hypothetical mediator of the correlation between *SCC* and *creativity rating score*. The mediation analysis revealed that the correlation between *SCC* and *creativity rating score* could potentially be mediated by the *BFI-O* scores, as we observed a loss of significance of the direct path when including the mediator effect (linear regression of *SCC* against *creativity rating score* (without the mediator): β_*SCC*–_*_*creativity rating score*_* = 0.68, *p* = 0.009 [*F*(1, 284) = 7.05, *p* = 0.008, *R*^2^ = 0.024]; linear regression of *SCC* against *creativity rating score* and *BFI-O* (the mediator): β*_SCC–_*_*creativity rating score*_* = 0.37, *p* = 0.20; *F*(2, 283) = 6.23, *p* = 0.002, *R*^2^ = 0.042]. The difference between β_*SCC*–_*_*creativity rating score*_* and β*_SCC–_*_*creativity rating score*_* was confirmed using bootstrapping [95% Confidence Interval CI = (0.010.62)]. Since our main aim was to assess the creativity change related to the lockdown, we decided to use the *SCC* score as our main variable of interest, as *creativity rating score* seems to reflect more the creative personality traits than the change in creativity during the lockdown period, and because baseline measurement of *creativity rating score* was not available.

#### Relations Between *Subjective Creativity Change* and Subjective Experience During the Lockdown

We explored the link between *SCC* and the variables reflecting the living conditions and subjective experience of the lockdown situation. To reduce our set of variables into a smaller set of dimensions, we conducted a PCA on the ten variables of interest using the available complete data from 293 participants (see section “Materials and Methods”). The Kaiser–Meyer–Olkin measure was 0.75, above the acceptable limit of 0.5. Three factors had eigenvalues over one and, in combination, explained 56.48% of the variance. Figure 1B shows the correlation matrix between variables, and Table 2 shows the components loadings after rotation. The variables that cluster on the same component suggest that component 1 represents affective changes during the lockdown, component 2 represents changes in the available time, and component 3 interactions with other people.

**TABLE 2:** Summary of exploratory principal component analysis results for the variables hypothetically related to creativity changes during the lockdown period (*n* = 293).

	Component 1	Component 2	Component 3
	Affective change	Available time	Interactions
R Anxiety and stress	**0.72**	0.12	−0.28
Mood	**0.82**	0.13	0.00
Motivation	**0.70**	0.09	0.13
R Pressure	**0.57**	**0.55**	-0.19
R Loneliness	**0.68**	−0.12	0.23
R Physical constraints	**0.46**	−0.31	−0.25
R Working hours	−0.11	**0.77**	0.03
Free time	0.27	**0.71**	−0.02
Space per cohabitant	0.20	−0.21	−**0.49**
Social interactions	0.34	−0.20	**0.81**
Eigenvalues	3.036	1.527	1.086
% of variance	30.36	15.27	10.86

*Loadings greater than 0.4 are in bold.*

We searched for correlations between the participant’s components loadings and *SCC*. *SCC* correlated significantly with component 1 representing affective changes during lockdown [*r*(291) = 0.30, *p* < 0.001] and with component 2 representing changes in time available [*r*(291) = 0.12, *p* = 0.033], but not with component 3 representing interactions with other people [*r*(291) = 0.003, *p* = 0.96] (Figure 1C). Individual correlations of each variable and *SCC* are provided in Supplementary Table 4. These results indicate that two main factors were associated with *SCC* during the lockdown period: affective changes and change in the available time. Restrictions in social interactions were not significantly associated with creativity changes.

As creativity could help cope with difficult situations and regulate emotions, we first explored whether creative personality traits could be linked to the subjective and affective experience of the lockdown. We found a significant correlation between *BFI-O* and component 1 (representing affective changes during lockdown) [*r*(291) = 0.19, *p* < 0.001]. Then, to better understand how *BFI-O* could have impacted both changes, we conducted two mediation analyses with either component 1 (PC1) as a mediating variable of the relationship between *BFI-O* and *SCC* (Mediation PC1), or *SCC* as a mediating variable of the relationship between *BFI-O* and component 1 (Mediation *SCC*). The results of Mediation *SCC* showed a significant partial mediation with a decrease in the direct path when including the mediator effect (linear regression of *SCC* against *BFI-O* (without the mediator): β_*BFI*–*O*–*PC*1_ = 0.03, *p* < 0.001; *F*(1, 291) = 11.37, *p* < 0.001, *R*^2^ = 0.038; linear regression of PC1 against *SCC* and *BFI-O*: β*_BFI–*O*–*PC*1_ = 0.02, *p* = 0.02; *F*(2, 290) = 17.80, *p* < 0.001, *R*^2^ = 0.109). The difference between β_*BFI*–*O*–*PC*1_ and β*_BFI–*O*–*PC*1_ was confirmed using bootstrapping [95% CI = (0.005 0.018)]. The results of Mediation PC1, did not reveal any significant mediation of BFI-O and *SCC* by PC1 [CI = (−0.13 0.29)]. Hence, individuals with higher openness may potentially had a better affective experience partially because they have felt more creative to during the lockdown.

Regarding demographic and other categorical factors, there was no significant effect of gender [*F*(1, 378) = 0.01, *p* = 0.93], age [*r*(378) = −0.02, *p* = 0.69], education [rho(378) = 0.04, *p* = 0.44], socio-professional category [*F*(8, 350) = 1.45, *p* = 0.17], socio-professional domain [*F*(19, 339) = 0.64, *p* = 0.88], or residential area [*F*(2, 377) = 0.43, *p* = 0.65] on *SCC* (see Table 1). Being at home [*F*(1, 378) = 0, *p* = 0.99], having a job related to COVID-19 [*F*(1, 378) = 0, *p* = 0.99], or working remotely [*F*(1, 378) = 0.56, *p* = 0.46) had also no significant association with *SCC*.

### Change in Creative Activities

In the survey, we qualitatively and quantitatively assessed the creativity change experienced during the lockdown. In total, 343 participants were presented with 28 activities (among them, 37 participants completed an older version of the questionnaire that included additional questions not considered here). All activities are reported in Supplementary Table 2. The 28 activities were selected based on existing items in other validated creativity questionnaires (see section “Materials and Methods”) and were framed in terms of creative activities, with phrasing such as “I created …,” or “I invented ….” For each of them, participants were first asked to indicate whether they had performed the activity during the last 5 years (lockdown included). If so, they were asked whether they did it during the lockdown and how often (more or less than before), for which reasons (various reasons were proposed for negative and positive frequency changes), and how they valorized it. For each activity that was less performed during the lockdown than before, the participants were asked whether they thought more or less about it during the lockdown.

#### Activities

**FIGURE 2:**
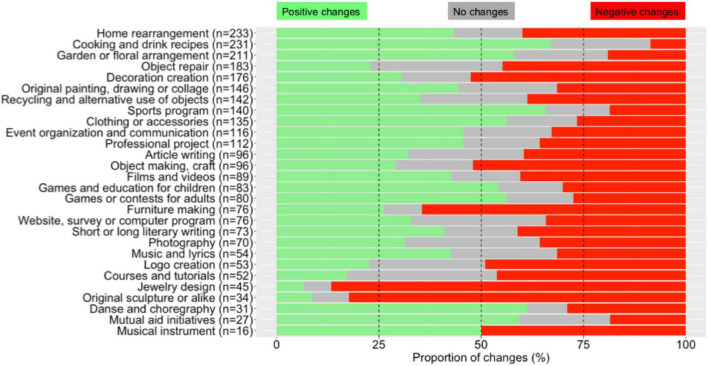
Frequencies and changes in activities. Proportions of changes across subjects regarding each of the 28 activities on the list for positive changes (in green), negative changes (in red), or no changes (in gray). Changes refer to a difference reported by each subject between the five last years preceding the lockdown and the lockdown period. Each activity is named by its English-translated quick name (see Supplementary Table 4) and is associated with the number of subjects who answered this question (i.e., subjects who did the activity during the last 5 years, including the lockdown period). Activities are sorted in descending order based on the number of subjects involved in this activity.

We identified activities that were relevant at the participant level to further estimate a lockdown-related change. On average, participants engaged in 8.38 ± 0.25 (SEM) activities (out of the 28 proposed) during the previous 5 years (including the lockdown period). Three participants did not engage in any activity, and four participants engaged in more than 20 activities. All the activities were performed at least by 9.9% of the participants during the lockdown (Figure 2). The activities of our list were consistent with the top-productions that the participants freely reported (see “Top-production” section below).

We also quantified the changes that occurred during the lockdown for each activity performed in the last 5 years (Figure 2). Among the activities performed during the last 5 years, the five activities with the highest increase in frequency during the lockdown across participants were “Cooking and drink recipes” (67.1% of the participants, *n* = 233), “Sports program” (65.7%, *n* = 140), “Dance and choreography” (61.3%, *n* = 31), “Mutual aid initiatives” (59.3%, *n* = 27) and “Garden or floral arrangement” (58.1%, *n* = 211). Activities with the deepest decrease in frequency during the lockdown were “Jewelry design” (86.7%, *n* = 45), “Original sculpture or alike” (82.4%, *n* = 34), “Furniture making” (64.5%, *n* = 75), “Decoration creation” (52.5%, *n* = 176), and “Object making, craft” (52.1%, *n* = 96).

#### Quantitative Changes in Activities During the Lockdown Period

**FIGURE 3:**
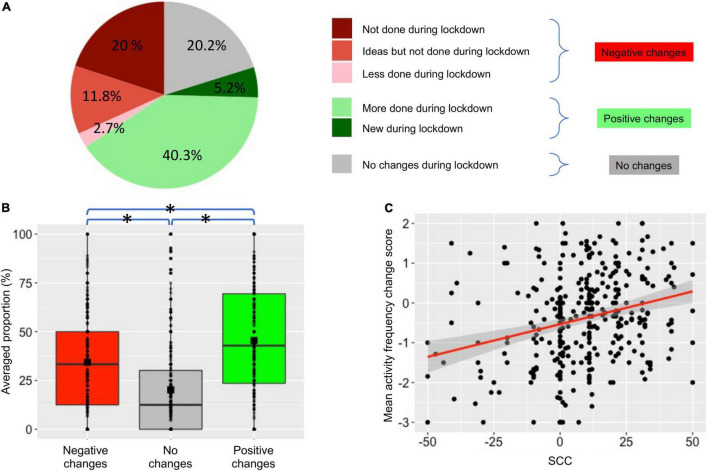
Quantitative changes in performing creative activities during the lockdown period. **(A)** Averaged proportions of changes across participants in performing activities. We show the activities with negative changes, positive changes, and no changes separately. The activities with negative changes were not done during the lockdown (dark red), were not done, but participants had ideas to do it (light red) or were done less frequently than before the lockdown (pink). The activities with positive changes were done more frequently (light green) or were new (dark green) during the lockdown. The activities with no changes were done as frequently (gray) as before the lockdown. **(B)** Averaged proportion of changes across participants for negative changes (red), no changes (gray), and positive changes (green) during the lockdown period compared to the five last years. Each dot represents a participant, color boxes represent the upper and lower quartiles, and squares indicate the mean. Stars indicate *post-hoc* tests with a p_*corr*_ < 0.05. **(C)** Participant’s *SCC* as a function of the averaged *activity frequency change score* (a dot per participant). The red line indicates a significant correlation between *SCC* and the mean *activity frequency change score* (Spearman correlation, *p* < 0.05).

To quantify the overall changes in performing creative activities during the lockdown (based on our list of 28 activities), we computed an *activity frequency change score* for each subject (Figure 3A). On average, 40.3 ± 1.5 (SEM)% of the activities performed by the participants during the last 5 years were carried out more frequently during the lockdown, and additionally, 5.2 ± 0.7% of the activities were new ones. Conversely, 20.2 ± 1.3% were carried out at the same frequency, 2.7 ± 0.4% less frequently, and 20 ± 1.2% were not performed at all during the lockdown. Another 11.8 ± 0.2% of the activities were not performed, but the participants thought about it during the lockdown period. The proportions of activities that were more (positive changes), less (negative changes), and equally (no change) performed during the lockdown differed significantly [*F*(2, 678) = 49, *p* < 0.001 with Greenhouse–Geisser correction; Figure 3B]. *Post hoc* Tukey tests revealed significant differences between all pairwise comparisons (p_*corr*_ < 0.001). Importantly, the mean *activity frequency change score* was significantly correlated with *SCC* [rho(329) = 0.23, *p* < 0.001; Figure 3C].

#### Reasons and Motives

**FIGURE 4:**
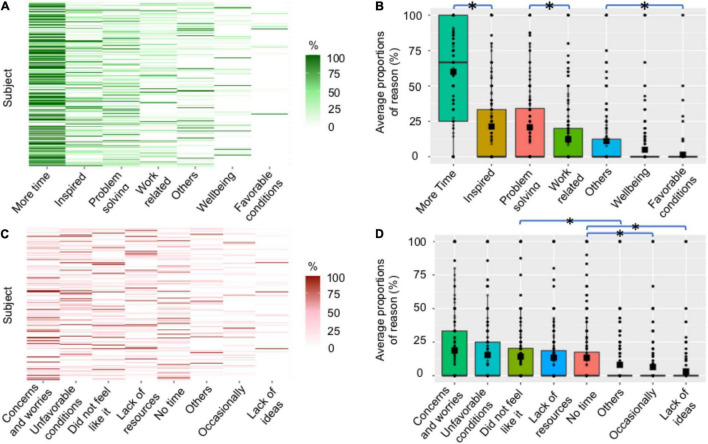
Reported reasons for positive and negative changes in creative activities. **(A)** Proportions of reasons for positive changes. Each row represents a participant, and reasons are sorted by decreasing average proportion across subjects. **(B)** Boxplots of the average proportion of reasons for positive changes across subjects. Each dot represents a participant, color boxes represent the upper and lower quartiles, and squares indicate the mean. Stars indicate significant differences between proportions of selected reasons (*post-hoc* tests with p_*corr*_ < 0.05). **(C,D)** Are the equivalent of **(A,B)**, respectively, for negative changes.

To examine why each activity was more or less frequently performed during the lockdown than before, we analyzed the reasons provided by the participants for positive and negative changes separately (Figure 4). Note that they could select several reasons for each activity during the survey. Across all activities, the frequency with which the different reasons for positive changes were selected significantly differed [*F*(6, 1,722) = 152, *p* < 0.001 with Greenhouse–Geisser correction; Figures 4A,B].

The most frequently selected categories of reasons for positive changes were: (1) having more free time (“More time,” 60 ± 2.3 (SEM)% of the activities carried out by each subject); free time significantly differed from each of the other reasons (p_*corr*_ < 0.05 for all *post-hoc* tests); (2) being inspired by the lockdown situation (“Inspired,” 21.3 ± 1.7%); and (3) having to solve lockdown/pandemic issues (“Problem solving,” 20.8 ± 1.6%). The frequency of those two last reasons did not statistically differ but was higher than the frequency of each of the remaining reasons (p_*corr*_ < 0.05 for all corresponding *post-hoc* tests). Additionally, 12.3 ± 1.3% of the activities performed more often than before were carried out for work (“Work related”), and 11.1 ± 1.3% for other undetermined reasons (“Others”). The less impactful reasons were to feel better (“Wellbeing”) and that the conditions of lockdown were favorable to this activity (“Favorable conditions”) (p_*corr*_ < 0.05 for all *post-hoc* tests, when compared to the other reasons).

Among the reasons for negative changes, the frequency of the reported categories of reasons significantly differed [*F*(7, 1925) = 14, *p* < 0.001 with Greenhouse–Geisser correction; Figure 4D]. The most selected reasons for carrying out an activity less frequently than before were: (1) having other concerns or worries (“Concerns and worries,” 18.8 ± 1.7%), (2) not having favorable conditions (“Unfavorable conditions,” 15.3 ± 1.6%), (3) not willing or not feeling like doing it (“Did not feel like it,” 14.1 ± 1.6%), (4) lacking the necessary material to do it (“Lack of resources,” 13.1 ± 1.5%), (5) not having enough time to do it (“No time,” 13.3 ± 1.6%). The frequency of those five main reasons did not statistically differ from each other (p_*corr*_ > 0.05 for all *post-hoc* tests). However, the frequency of each of these reasons differed significantly from the remaining reasons that were: not having ideas for this activity (“Lack of ideas,” 3 ± 0.8%), that this activity was performed only occasionally before and was not common for the participant (“Occasionally,” 6.3 ± 1.1%), or other undetermined reasons (“Others,” 7.9 ± 1.2%) (p_*corr*_ > 0.05 for all *post-hoc* tests).

To check the consistency of the participant’s responses to the first part of the survey (global experience of the lockdown) and the second part (activity-based questions), we explored whether the activity-based reported reasons correlated with the global subjective experience of the lockdown. The difference between selecting “Inspired” for positive reasons and “Concerned and worries” or “Did not feel like it” for negative reasons was correlated with Component 1 (reflecting affective experience) across individuals [*r*(280) = 0.12, *p* = 0.04]. Additionally, we found that the difference between selecting “More time” for positive reasons and “No time” for negative reasons was correlated with Component 2 (reflecting more free time) across individuals [*r*(280) = 0.51, *p* < 0.001] (see section “Materials and Methods” and Supplementary Figure 2).

#### Obstacles

For each activity carried out more frequently or as often as before the lockdown, participants were asked how many obstacles they had to overcome to conduct this activity (on a scale ranging from 0 to 100). The overall mean *obstacle score* was 15.44 ± 0.79 (SEM). The mean *obstacle score* was not significantly correlated to *SCC* [*r*(329) = −0.04, *p* = 0.49]. However, using a polynomial fit of second-order, we identified a quadratic relationship between those two measures [β_*quadr*_ = 0.004 ± 0.001 (SE), *t*(328) = 2.82, *p* = 0.005; β_*lin*_ = −0.07 ± 0.04, *t*(328) = −1.56, *p* = 0.12; *F*(2, 328) = 4.26, *p* = 0.015, *R*^2^ = 0.025] (Supplementary Figure 3), suggesting that the amount of encountered obstacle was high for individuals experiencing a large negative or positive *SCC*.

#### Creative Achievements

**FIGURE 5:**
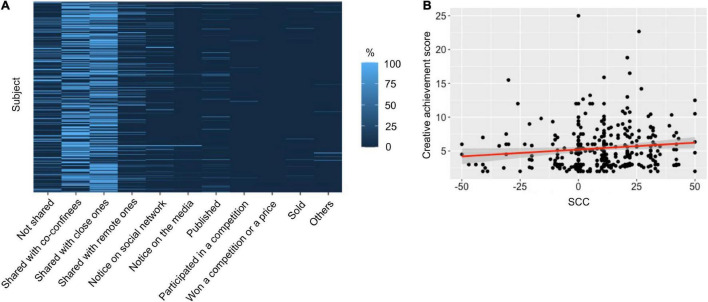
Creative achievements. **(A)** Proportion of the selected levels of creative achievement averaged across activities for each subject. **(B)** Individual *SCC* as a function of individual *creative achievement score*. The red line indicates a significant correlation between *SCC* and the *creative achievement score* (Spearman correlation, *p* < 0.05).

In this survey, we estimated, for each participant, the level of creative achievements for the activities performed during the lockdown by adapting items of an achievement questionnaire (ICAA, part 2; Diedrich et al., 2017; Figure 5 and Supplementary Table 3 for details). For the activities carried out more frequently or as often during the lockdown as before, we asked the participants to report how they valorized their activities. The participants reported that for 53.98 ± 1.80% of the activities they carried out, they shared the result with family, friends, or colleagues, and 16.96 ± 1.47% were shared with no one. For 11.08 ± 1.09% of the performed activities, the results were communicated to the participant’s remote social network. Among higher achievements, the participants reported that the result of their activity was noticed outside of their usual network for 5.89 ± 0.81% of their activities, published for 4.28 ± 0.66%, or mentioned in the media for 1.27 ± 0.43%. Other achievements represented less than 1% of the activities each. Importantly, based on the level of achievement reported by the participants, we computed a *creative achievement score* per participant (see “Materials and Methods”), and we found that it was significantly correlated with *SCC* [rho(298) = 0.16, *p* = 0.004].

### Top-Productions

At the end of the survey, participants were asked to freely report their most creative productions (up to five) during the lockdown. To check the validity of our list of activities, we examined whether each of the 1,091 reported top-productions corresponded to an activity included in our list. We found that 90% of the top-productions reported by the participants were in the list (with a M ± SEM *creativity rating score* of 2.1 ± 0.03 on a rating scale from 0 to 5). Moreover, among the 10% of the top-productions that were not referring to an activity included in our list, 83.5% had a *creativity rating score* lower than 2.5 (with a M ± SEM *creativity rating score* of 1.3 ± 0.1). The majority of those low-rated top-productions were about “learning something new,” “storing/classifying,” “reading,” and “projects/ideas.” Among the top-productions not covered by our list, the top-productions with a score higher than 2.5 (18 productions) were about ecology (*n* = 3), artistic projects or ideas (*n* = 4), and diverse multimedia creations (*n* = 3).

To analyze the frequency of the productions among the participants, we recoded the name of the top-productions and selected the ones with a mean *creativity rating score* higher than 0.5. “Cooking and recipes” were the most frequent realizations (reported by 31% and 19% of the participants), followed by “Writing” (25%), “Manufacturing” (19%), “Gardening” (18%), and “Sewing” (13%).

## Discussion

Using an online survey, we aimed to understand the impact of the COVID-19-related lockdown in French-speaking population on creativity and the main factors explaining this impact. Our main *a priori* factors were the affective states, available time, personal conditions of lockdown, and social isolation. Our survey used two main approaches to quantify individual creativity changes: subjective self-rated creativity changes (*SCC*), and activity-based measures. Our study documents the unprecedented situation we all had to face during the lockdown and sheds new lights on potential factors that could have modulated individual creative behavior.

### Creativity During the Lockdown

*SCC* analyses revealed that participants experienced being more creative during the lockdown than the period before, which is consistent with previous studies that reported an increase in everyday creativity during the lockdown (Karwowski et al., 2021; Mercier et al., 2021; Morse et al., 2021). Lockdown-related creativity changes using the activity-based questionnaire approach were also consistent with the *SCC* report. Notably, both the *activity frequency score* and the *creative achievement score*, correlated with *SCC*, indicating that *SCC* related to changes in creative activities and achievements. We also found a quadratic relationship between the amount of obstacles to overcome to achieve an activity assessed by activity-based questionnaire (*obstacle score*) and *SCC*: individuals who declared having a decrease in their creativity had to face too many obstacles while individuals experiencing an increase in their creativity might have been stimulated by the amount of obstacles. This result may suggest that creative behaviors differed according to the perception or the nature of an obstacle. Individual factors that influence our creative behavior in facing obstacles remain to be explored. As the *SCC* came early in the survey, before asking participants about the activities they engaged in during the lockdown, it is unlikely that activity-based questions influenced our *SCC* measure (Karwowski et al., 2019).

In addition, *SCC* correlated with the *BFI-O* score, which captures a basal creative personality trait (Jauk et al., 2014; Beaty et al., 2017), openness, and which was in the range previously observed (Denissen and Penke, 2008). The linear relationship between *SCC* and *BFI-O* indicates that individuals with higher openness to new experiences reported proportionally more positive self-rated creativity changes. Measures of creative confidence beliefs, such as self-rated creativity, classically correlate with openness (Karwowski and Lebuda, 2016), which is considered as a big five personality trait that impacts creative self-efficacy and self-concept.

We also assessed creativity during the lockdown using an external rating approach of the top-5 creative productions freely reported by the participants (*creativity rating score*). *SCC* correlated positively with this measure, indicating that the participants who reported a higher increase in their creativity also had higher creativity ratings of their top-productions. Interestingly, *BFI-O* mediated the relationship between *SCC* and *creativity rating score*, suggesting that creativity during the lockdown (i.e., external ratings of the participants’ productions) depended on both a basal creative personality trait (*BFI-O*) and the subjective feeling of lockdown-related changes in creativity (*SCC*).

Overall, these findings indicate that our self-rated measure *SCC* relates to creative personality traits and several creativity measures based on real-life activities and achievements, in accordance with the model of primary and secondary creativity (Runco and Beghetto, 2019) and can capture event-related changes in creativity. *SCC* may be an interesting measure of real-life creative changes that remains to be validated in future cognitive studies measuring changes with baseline comparison.

### Activities Carried Out During the Lockdown: Quantitative and Qualitative Aspects

Our activity-based questionnaire offered an overview of the participants’ engagement in creative activities during the lockdown and related changes using a list of 28 predefined activities. We also asked them to freely report their five most creative productions during the lockdown. Overall, 90% of the self-reported top-productions corresponded to activities included in our list, suggesting that our list was indeed representative of the activities performed during the lockdown period. “Home rearrangement,” “Cooking and drinks recipes,” and “Garden or floral arrangement” were the three activities in which participants engaged the most. These creative activities were consistent with a pre-pandemic study that assessed the frequency of creative activities in several domains (Benedek et al., 2019).

The activity-based analysis revealed a global quantitative increase in carrying out creative activities during the lockdown, which is consistent with the increase in self-rated creativity shown by the *SCC* results. The activities with the highest increase in frequency were “Cooking and drinks recipes,” “Sports program,” “Dance and choreography,” “Mutual aid initiatives,” and “Garden and floral arrangement.” This result echoes studies in other countries showing that individuals increased their cooking and baking activities (Gerritsen et al., 2020), physical training (Di Renzo et al., 2020) during the lockdown. For the explored activities, the *creative achievement score* of each subject was relatively low, indicating that our questionnaire assessed “everyday creativity” rather than extraordinary achievements. Nevertheless, the significant correlations of the *activity frequency change score* and *SCC*, combined with the fact that top productions of the participants broadly corresponded to activities in our list, suggest that our activity-based measure captured not only the quantity of activities but also likely their creativity.

### Potential Reasons for the Impact of the Lockdown Situation on Creativity

We explored how several factors could be related to creativity changes. No demographic factors correlated with the *SCC*. However, using PCA, we found that the two first principal components of the lockdown conditions and subjective experience significantly correlated with *SCC*. The first component that we labeled “affective change” included *anxiety and stress*, *motivation*, psychological *pressure*, *mood*, and to a lower extent, *loneliness* and *physical constraints*. The relationship between this first component and *SCC* is consistent with a series of studies showing that affective states such as positive mood and motivation are associated with higher creativity (Collins and Amabile, 1999; Baas et al., 2008) and that intrinsic motives drive everyday creative activities (Benedek et al., 2019). Conversely, negative affective states hindered creativity in some individuals. This result is also consistent with a recent studies showing a relationship between positive affective states during the lockdown and creativity (Elisondo, 2021; Hofreiter et al., 2021). Our survey shows that overall, our participants reported more negative subjective and affective experiences during this period than before. This finding converges with other studies conducted worldwide during the lockdown of Spring 2020 that showed that people experienced an increase in stress and depression symptoms in Italy (Roma et al., 2020), Spain (Ozamiz-Etxebarria et al., 2020), New Zealand (Every-Palmer et al., 2020), Kuwait (Burhamah et al., 2020), and Croatia (Ðogaš et al., 2020). Interestingly, we found a significant mediation effect of *SCC* on the relationships between *BFI-O* and the first principal component (reflecting affective changes). This result suggests that higher openness may have facilitated creativity expression in during the lockdown, which in turn may have help to cope with difficult psychological situations (Wu et al., 2019). Hence, creative traits and abilities may be essential to resilience. Note that significant mediation effects does not mean that we identified a true causal effect, instead, it places some variables as candidates for causal mediators, that should be experimentally addressed (Fiedler et al., 2011, 2018).

The second principal component that correlated with *SCC* reflected the available time, including the *number of working hours*, *free time*, and to a lower extent, *space per cohabitant*. An increase in free time was linked to higher creativity change. This finding is in agreement with studies that investigated the time pressure as hindering creativity (Amabile et al., 1996, 2002). Note that the strength of correlation between SCC and the second principal component (change in available time) was not as strong as the correlation with the first principal component (change in affective state), suggesting that the available time did not impact as much as the affective change on creativity during the lockdown.

It is important to mention that given the correlational nature of the relationship between change in creativity and affective change, we cannot conclude about any causality effect: more positive affective states could trigger higher creativity, but the reverse could be true, or another unknown variable could mediate this correlation. However, the coherence of our findings based on two different sets of measures is to be highlighted. In addition to a global subjective report of their self-perceived emotions, free time, and creativity, the participants indicated the reasons why they achieved or gave up specific creative activities during the lockdown. The reasons for carrying out an activity more frequently were dominated by more free time, while the reasons for carrying out an activity less frequently were mainly related to concerns and worries. The individual proportion of these reasons correlated with the individual component loadings of the two first principal components (reflecting affective state and free time) identified among the subjective experience factors that we collected. These results indicate strong consistency between the different parts of our questionnaire.

Overall, the correlations of *SCC* with the reported affective and free time changes on the one hand, and the identified reasons for engaging more or less frequently in creative activities during the lockdown than before on the other, converge to the crucial role of emotions and free time on creativity during this period. The exact nature and context of the affective factors remain to be clarified.

### Limitations

One potential limitation of our study is that our sample is not fully representative of the French population. For instance, our sample had more women, with a high education level, and most of them could work remotely. It is also possible that individuals who participated in this survey were inherently interested in creativity or had more time for online surveys. Thus, the increase of creativity observed in this sample might not be replicable in other populations, such as individuals who could not work remotely or had no time to answer online surveys. In addition, the participants responded just after the end of the lockdown, their reports are thus retrospective and may lack accuracy or objectivity. Yet, it is worth mentioning that most surveys used questions regarding the actual lockdown period, with difficulties in measuring a valid baseline, while in our study, we focused on the change between before and during the lockdown.

## Conclusion

The massive lockdown provoked by the COVID-19 pandemic gave an exceptional opportunity to investigate how various factors and exceptional situations could impact everyday creativity. Our study documents this unique situation regarding creativity, creativity enablers, and blockers based on the description of how individuals experienced this situation and how they adapted their everyday creative activities. Our subjective report of self-rated creativity and activity-based questionnaire provide converging results supporting an overall positive change in creativity during the lockdown period, which was mainly related to having more free time or opportunities. However, the changes were heterogeneous across participants, and when negative changes in self-rated creativity or creative activities were reported, they were mainly related to negative affective factors. A creative personality may have partially helped to better express one’s creativity, and to cope with the negative emotions induced by the crisis. Those factors have thus to be considered when trying to enhance creativity or well-being in individuals and society. Our observations may provide interesting hypothesis to explore in future studies for a better understanding of the affective, emotional, and wellbeing dimensions that modulate our creativity, which could inspire political, societal or management decisions.

## Data Availability Statement

The datasets presented in this study can be found in online repositories. The names of the repository/repositories and accession number(s) can be found below: https://osf.io/XBR78/. A Matlab Application is also available to explore the dataset and reproduce all results presented in the article.

## Ethics Statement

The studies involving human participants were reviewed and approved by Comité d’Evaluation Ethique de l’INSERM. The participants provided their written informed consent to participate in this study.

## Author Contributions

TB, MO-T, EV, and AL-P performed the data analyses and wrote the manuscript. AL-P designed the open MatLab App. All authors designed the online survey, collected the data, discussed the results, and commented the manuscript.

## Conflict of Interest

The authors declare that the research was conducted in the absence of any commercial or financial relationships that could be construed as a potential conflict of interest.

## Publisher’s Note

All claims expressed in this article are solely those of the authors and do not necessarily represent those of their affiliated organizations, or those of the publisher, the editors and the reviewers. Any product that may be evaluated in this article, or claim that may be made by its manufacturer, is not guaranteed or endorsed by the publisher.
