# Supplementary material for: Through Thick and Thin: Changes in Creativity During the First Lockdown of the COVID-19 Pandemic
Source: Front Psychol. 2022 May 10;13:821550. doi: 10.3389/fpsyg.2022.821550 (PMC9127054; doi:10.3389/fpsyg.2022.821550)
Supplement: Supplementary file 1 [file Data_Sheet_1.docx]

## Supplementary material

## Supplementary method 1:

Description of the survey

In our survey, we used independent question distributed into nine distinct sections in the following order (n indicate the number of included participants who responded to each section):

1. Demographic information, including age, gender, education level (n=380)
2. Condition of lockdown, including residential area type, own residence, access to private or large public outdoor spaces, number of cohabitants and the number of available rooms in the residence place, whether professional activity was COVID-19 related, professional situation, the occurrence of a serious problem that could have limited the initiation of activities (and in this case, how long it limited one’s activities), changes in social interactions, change in the amount of free time (n=380).
3. Affective experience, including change in physical constraints, loneliness, mood, motivation, anxiety and stress, and pressure (n=380).
4. Subjective creativity change measured as the SCC (n=380).
5. BFI-O (n=380).
6. The participant’s behavior regarding a list of 28 creative activities (n=343).
7. Their ideational behavior regarding a list of three topics. This part was not included in the current study, which focuses on activities instead of ideation (n=343).
8. Complementary information about demographics and lockdown situation including socio-professional activity (n=359), main field of activity (n=359), the number of working hours per week (n=358, with 65 participants not concerned, considered n=293), and the country of residence (n=323).
9. The top-creative productions they carried out during the lockdown (n=286).

## Figure S1

**Supplementary Figure S1.** Schematic representation of the conditional sequence of questions proposed for each activity

## Figure S2

**Supplementary Figure S2.** Correlations between activity-based reported reasons (the difference between positive and negative reasons) and the subjective components related to creativity changes measured in the first part of the survey. Left: Proportion of ‘Inspired’ minus proportion of ‘Concerned and worries’ and ‘Did not feel like it’ selected respectively as positive and negative reasons for carrying out an activity per individual as a function of component loading of affective change. Right: Proportion of ‘More Time’ minus the proportion of ‘No time’ selected respectively as positive and negative reasons for carrying out an activity per individual as a function of component loading of available time.

## Figure S3


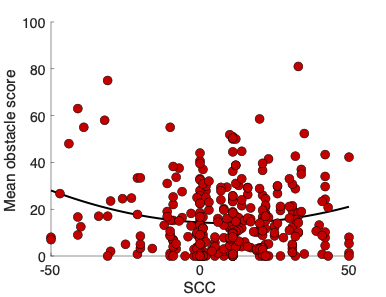


**Supplementary Figure S3.** Individual mean obstacle score across activities as a function of individual SCC. The solid line represents a second-order polynomial fit, illustrating a significant quadratic link (p<0.05).

## Table S1

Additional demographic information, with statistical tests (one-factor ANOVA) on SCC. SCC mean and SEM are reported in the table. N indicates the number of participants in each category. No statistical test was performed when one group represented less than 5% of the data (in italic).

| Participants | | Total included | | SCC Mean | SCC SE |
| --- | --- | --- | --- | --- | --- |
|  |  | 380 | | 8.0 | 0.98 |
|  | | | | | |
| Professional Activity  (F(1, 378)=0, p=0.99) | | N | % | SCC Mean | SCC SE |
| Related to the fight against COVID-19 | | 43 | 11.32 | 7.86 | 3.15 |
| Unrelated to the fight against COVID-19 | | 315 | 82.89 | 7.87 | 1.06 |
|  | | | | | |
| Socio-professional category  (F(8, 350)=1.45, p=0.17) | | N | % | SCC Mean | SCC SE |
| Agriculture/Operator | | 2 | 0.53 | 17 | 17 |
| Artisan/ Merchant/Entrepreneur | | 4 | 01.05 | 12 | 15.95 |
| Professionals and intellectual professions | | 162 | 42.63 | 8.44 | 1.37 |
| Intermediate professions | | 28 | 7.37 | 09.07 | 3.66 |
| Employee | | 49 | 12.89 | 11.88 | 2.6 |
| Student | | 43 | 11.32 | 6.93 | 2.7 |
| Salaried worker | | 1 | 0.26 | 33 | 0 |
| Retired | | 33 | 8.68 | 5.62 | 2.95 |
| Other | | 37 | 9.74 | -0.3 | 4.64 |
|  | | | | | |
| Main field of activity  (F(19,339)=0.64, p=0.88) | | N | % | SCC Mean | SCC SE |
| Agriculture / Agronomy | | 3 | 0.79 | 15 | 10.02 |
| Arts/ Design/ Architecture | | 29 | 7.63 | 5.97 | 5.09 |
| Audiovisual / Cinema / Spectacle/ Musician | | 11 | 2.89 | 5.45 | 5.4 |
| Audit / Accounting | | 8 | 2.11 | 7.75 | 7.09 |
| Building / public worker | | 2 | 0.53 | 6.5 | 6.5 |
| Food trade / Distribution | | 1 | 0.26 | 20 | 0 |
| Non - food retail / Distribution | | 6 | 1.58 | 0.17 | 11.4 |
| Culture / Artisan | | 13 | 3.42 | 2.54 | 7.11 |
| Law/ Justice/ Economy / Management | | 9 | 2.37 | 18.11 | 6.2 |
| Education | | 25 | 6.58 | 10.04 | 3.51 |
| Finance/ Bank/ Insurance | | 12 | 3.16 | 5.58 | 7.03 |
| Industry | | 6 | 1.58 | 9.83 | 9.67 |
| Computer science / Engineering | | 28 | 7.37 | 3.29 | 2.65 |
| Medias/ Communication / Marketing / Advertising | | 17 | 4.47 | 14.53 | 3.79 |
| Research / Higher Education | | 69 | 18.16 | 8.46 | 1.97 |
| Health | | 70 | 18.42 | 8.46 | 2.27 |
| Security | | 0 | 0 | - | - |
| Social | | 21 | 5.53 | 7.48 | 3.76 |
| Sports / Leisure | | 6 | 1.58 | 4.17 | 11.93 |
| Tourism / Hotel / Restoration | | 3 | 0.79 | 9.67 | 6.33 |
| Transportation | | 0 | 0 | - | - |
| Other | | 20 | 5.26 | 9.1 | 3.16 |
|  | | | | | |
| Professional situation during confinement | | N | % | SCC Mean | SCC SE |
| I kept moving to my workplace  (F(1, 356)=0.77, p=0.38) | Yes | 54 | 14.21 | 5.76 | 2.56 |
|  | No | 304 | 80.00 | 8.24 | 1.1 |
| Remote working  (F(1, 356)=0.56, p=0.46) | Yes | 204 | 53.68 | 8.52 | 1.25 |
|  | No | 154 | 40.53 | 7 | 1.66 |
| Partial unemployment  (F(1, 356)=1.32, p=0.25) | Yes | 49 | 12.89 | 10.78 | 2.92 |
|  | No | 309 | 81.32 | 7.4 | 1.07 |
| Searching for a job  (F(1, 356)=0.8, p=0.37) | Yes | 31 | 8.16 | 4.94 | 4.02 |
|  | No | 327 | 86.05 | 8.14 | 1.04 |
| Student (F(1, 356)=0.53, p=0.47) | Yes | 41 | 10.79 | 5.83 | 3.15 |
|  | No | 317 | 83.42 | 8.13 | 1.06 |
| House-husband / housewife | Yes | 12 | 3.16 | 11.58 | 7.5 |
|  | No | 346 | 91.05 | 7.74 | 1.01 |
| Retired  (F(1, 356)=0.78, p=0.38) | Yes | 41 | 10.79 | 5.39 | 3.03 |
|  | No | 317 | 83.42 | 8.19 | 1.07 |
| On sick leave | Yes | 10 | 2.63 | 24.4 | 7.21 |
|  | No | 348 | 91.58 | 7.39 | 1.01 |
| Maternity / paternity leave | Yes | 2 | 0.53 | 26.5 | 10.5 |
|  | No | 356 | 93.68 | 7.76 | 1.01 |

## Table S2

List of the 28 activities for which participants had to respond a series of question. Original French sentence, English translation and short name are reported for each of the activity.

| Activity | French | English translation | Short name |
| --- | --- | --- | --- |
| 1 | J’ai fabriqué un meuble | I made a piece of furniture | Furniture making |
| 2 | J’ai créé une décoration pour mon habitation | I created a decoration for my home | Decoration creation |
| 3 | J’ai organisé ou réorganisé une pièce de mon habitation | I have organized or reorganized a room in my home | Home rearrangement |
| 4 | J’ai entrepris du jardinage ou réalisé une composition florale | I started gardening or made a flower arrangement | Garden or floral arrangement |
| 5 | J’ai réparé seul un objet cassé | I repaired a broken object alone | Object repair |
| 6 | J’ai conçu un bijou | I designed some jewelry | Jewelry design |
| 7 | J’ai conçu ou revisité un vêtement ou un accessoire (couture, tricot, ...) | I have designed or revisited an item of clothing or an accessory (sewing, knitting, ...) | Clothing or accessories |
| 8 | J’ai recyclé ou détourné un objet (non mentionné plus tôt) pour lui donner une deuxième vie | I recycled or diverted the use of an object (not mentioned previously) to give it a second life | Recycling and alternative use of objects |
| 9 | J’ai conçu ou revisité une recette de cuisine, une boisson ou un cocktail | I designed or revisited a cooking recipe, a drink or a cocktail | Cooking and drink recipes |
| 10 | J’ai mis en place des jeux ludiques et/ou pédagogiques pour des enfants (loisirs ou école à la maison) | I have set up fun and / or educational games for children (leisure or home school) | Games and education for children |
| 11 | J’ai inventé ou organisé un jeu, un quizz ou un concours (pour des amis, famille, collègues...) | I invented or organized a game, a quiz or a contest (for friends, family, colleagues ...) | Games or contests for adults |
| 12 | J’ai élaboré un programme ou un exercice d’entraînement sportif | I have developed a sports training program or exercise | Sports program |
| 13 | J’ai réalisé un tableau, un dessin ou un collage original | I made an original painting, drawing or collage | Original painting, drawing or collage |
| 14 | J’ai réalisé une sculpture, une mosaïque, une poterie ou de la céramique | I made a sculpture, a mosaic, a pottery or a ceramic | Original sculpture or alike |
| 15 | J’ai créé un slogan ou un logo | I created a slogan or logo | Logo creation |
| 16 | J’ai réalisé ou j'ai joué dans une vidéo, un montage, un court ou long-métrage, ou un film d’animation | I made or acted in a video, a montage, a short or full-length film, or an animation film | Films and videos |
| 17 | J’ai réalisé des images photographiques avec des techniques originales | I made photographic images with original techniques | Photography |
| 18 | J’ai écrit une histoire, un poème, une nouvelle, un roman, une bande dessinée, ou une pièce de théâtre | I wrote a story, a poem, a short story, a novel, a comic book, or a play | Short or long literary writing |
| 19 | J’ai écrit un article | I wrote an article | Article writing |
| 20 | J’ai inventé ou revisité une musique, une mélodie, un rythme ou les paroles d’une chanson | I invented or revisited a music, a melody, a rhythm or the lyrics of a song | Music and lyrics |
| 21 | J’ai conçu un instrument de musique ou une manière de faire de la musique | I designed a musical instrument or invented a way to make music | Musical instrument |
| 22 | J’ai inventé ou ré-interprété une chorégraphie ou une danse | I invented or re-interpreted a choreography or a dance | Dance and choreography |
| 23 | J’ai conçu un programme informatique, un site internet ou une enquête | I designed a computer program, a website or a survey | Website, survey or computer program |
| 24 | J’ai créé un tutoriel ou un cours (hors sport et éducation des enfants) | I created a tutorial or a course (excluding sports and children's education) | Courses and tutorials |
| 25 | J’ai créé ou organisé un système ou réseau d’entraide (par exemple de distribution de nourriture ou de soutien aux personnes isolées) | I have created or organized a support system or network (for example for the distribution of food or support for isolated people) | Mutual aid initiatives |
| 26 | J’ai organisé de manière originale un événement (anniversaire, apéritif, ...) ou inventé des solutions alternatives pour échanger avec les autres (club, lettres, ...) | I organized an event in an original way (birthday, aperitif, ...) or invented alternative solutions to communicate with others (club, letters, ...) | Event organization and communication |
| 27 | J’ai fabriqué un objet ou un accessoire | I made an object or an accessory | Object making, craft |
| 28 | J'ai écrit un projet innovant ou diversifié mon activité dans le cadre de mon travail | I wrote an innovative project or diversified my activity as part of my work | Professional project |

## Table S3

Details of the presented options for reasons for change in the frequency of an activity, and details of the presented options of achievement reached for each activity. Original French sentence, English translation and short name are detailed. Ranking values were used to compute the achievement score.

|  | French | English translation | Short name | Ranking value |
| --- | --- | --- | --- | --- |
| Reasons for positive change | J'avais plus de temps | I had more time | More time | - |
|  | Le confinement et/ou l'épidémie m'ont inspiré.e | The situation inspired me | Inspired | - |
|  | Je devais résoudre un problème lié au confinement ou à l'épidémie de COVID-19 | I had to solve a problem related to the situation | Problem solving | - |
|  | Je devais le faire pour mon travail | I had to do it for work | Work related | - |
|  | Autres | Others | Others | - |
|  | Je l'ai fait pour me sentir mieux | I did it to feel better | Wellbeing | - |
|  | J'étais dans des conditions favorable pour le faire | I was in favorable conditions to do it | Favorable conditions | - |
| Reasons for negative change | Je n'ai pas eu le temps | I had no time to do it | No time | - |
|  | J'avais d'autre soucis ou préoccupations | I had other concerns | Concerns and worries | - |
|  | Mes conditions de confinement ne m'ont pas permis de le faire | I was not in favorable conditions to do it | Unfavorable conditions | - |
|  | Je n'avais pas les ressources matérielles nécessaires | I did not have the material opportunities to do it | Lack of resources | - |
|  | Je n'ai pas eu envie | I did not feel like it | Did not feel like it | - |
|  | Les idées ne me venaient plus | I hadn’t any ideas | Lack of ideas | - |
|  | Je ne l'avais fait que ponctuellement auparavant | I did it only a few times before | Occasionally | - |
|  | Autres | Others | Others | - |
| Levels of achievement | Je ne l'ai montré à personne | I did not share it | Not shared | 2 |
|  | Je l'ai montré à mes co-confinés | I shared it with my co-confinees | Shared with co-confinees | 3 |
|  | Je l'ai montré à mes amis, mes proches ou mes collègues | I shared it with my close ones | Shared with close ones | 3 |
|  | Je l'ai montré à des inconnus au-delà de mon cercle habituel ou sur les réseaux sociaux | I shared it with my remote ones | Shared with remote ones | 4 |
|  | Cela a été remarqué par des personnes au-delà de mon cercle habituel ou sur les réseaux sociaux | It was noticed on social networks | Noticed on social networks | 4 |
|  | Les médias en ont parlé | The media talked about it | Noticed on the media | 9 |
|  | Je l'ai publié | It was published | Published | 6 |
|  | Cela m'a permis de participer à un concours | I participated in a competition | Participated in a competition | 7 |
|  | Cela m'a permis de gagner une récompense ou un prix | I won a competition or a price | Won a competition or a price | 8 |
|  | J'ai réussi à le vendre | It was sold | Sold | 10 |
|  | Autre proposition | Other | Other | - |

## Table S4

Pearson correlation coefficients and p-values between SCC and living conditions / subjective experience of the lockdown situation. N indicate the number of participants included (available data).

|  | Pearson correlation coefficients with SCC | p-value (2-tailed) | N |
| --- | --- | --- | --- |
| R Anxiety and stress | 0.175 | 0.001 | 380 |
| Mood | 0.226 | <0.001 | 380 |
| Motivation | 0.462 | <0.001 | 380 |
| R Pressure | 0.185 | <0.001 | 380 |
| R Loneliness | 0.088 | 0.086 | 380 |
| R Physical constraints | 0.092 | 0.075 | 380 |
| R Working hours | 0.06 | 0.261 | 293 |
| Free time | 0.147 | 0.004 | 380 |
| Space per cohabitant | -0.069 | 0.242 | 380 |
| Social interactions | 0.079 | 0.125 | 380 |
